# Peer Review of “A Hybrid Pipeline for Covid-19 Screening Incorporating Lungs Segmentation and Wavelet Based Preprocessing of Chest X-Rays (Preprint)”

**DOI:** 10.2196/64675

**Published:** 2024-09-19

**Authors:** Daniela Saderi

**Affiliations:** 1 PREreview, Portland, OR, United States

**Keywords:** COVID-19, wavelets, convolutional neural networks, segmentation

*This is a peer-review report submitted for the preprint “A Hybrid Pipeline for Covid-19 Screening Incorporating Lungs Segmentation and Wavelet Based Preprocessing of Chest X-Rays.”*

This review is the result of a live review organized and hosted by PREreview and JMIR Publications on September 2, 2022. The call was joined by 15 people, including reviewers, preprint authors, and facilitators.

## Summary

The authors of this study [1] present a novel strategy and methodology to classify patients’ chest x-ray (CXR) images as SARS-CoV-2 positive (COVID-19 positive) and SARS-CoV-2 negative (COVID-19 negative). The proposed method is presented as an alternative to existing CXR evaluation methods, which require trained expertise and sophisticated equipment to be implemented. The methodology is based on a hybrid artificial intelligence pipeline dominantly using wavelet scattering processing for encoding the principal features of the CXRs. The reviewers indicated that this study is innovative and potentially very useful in identifying new positive cases associated with variants that escape reverse transcriptase–polymerase chain reaction–based diagnosis. The hybrid approach is a promising solution, particularly as it is presented as a quicker and more cost-effective solution for a pandemic that is here to stay. However, the reviewers raised some concerns and questions that they believe would be important for the authors to address. These are outlined below.

## Evidence and Examples: Concerns and Constructive Feedback

- The reviewers expressed some concern around the time in which the CXR images were taken across different patients, the severity of the cases, and other comorbidity factors that could have impacted the results. If that data is available, it would be helpful to compare CXRs across matched time points, as it is possible that different acuity phases of the infection lead to different results.
- The reviewers wonder if the fact that the contours of the region to analyze were identified by different radiologists may have potentially undermined the assessments of the model’s function. It would be useful to discuss the limitations of this approach in more depth.
- Module 2 was designed to test the replacement of learned coefficients with fixed coefficients; however, the alternative proposition of training a new network on the COVID-19 positive and negative CXRs was not explored. Are there implications that would need to be discussed related to this point?
- Can the model identify CXRs with lung infection and perhaps patients with acute respiratory distress syndrome? That is important to triage patients with COVID-19 for the risk of death.
- Can this same approach be used for the diagnosis of other pneumonia-like diseases? Is the model specific enough to differentiate true COVID-19 positive cases from other similar diseases that mimic SARS-CoV-2–induced lesions? Perhaps the authors can discuss the specificity of the model and its replicability a bit more.
- Have the authors checked the area under the curve values using, for example, ResNet50 or any other deep learning structure? In essence, does a variational autoencoder encode the same (or close) to the features encoded by the scattering wavelet approach?

Below are other general suggestions and recommendations to improve on the clarity of the work presented.

- Reviewers suggest rewriting the manuscript in the more classic “IMRD” format and having the Discussion section point out the limitations and overall conclusions of the study.
- Regarding figures, reviewers suggest the addition of headers to the figures to help the reader get oriented and better match the figure to the Results section.
- Reviewers found the figures of the “mask” used to identify contours of the portion of tissue analyzed very helpful and suggest the authors refer to that figure the first time they mention the mask in the text.
- Was the training dataset coming from a diverse group of individuals? If so, it would be great to know. If not, in the Discussion, it would be nice to see a reflection on the limitations that may arise from the fact that the data may come from a homogeneous group of people.
- In the Methods section please mention possible institutional review board exemption or approval and any other ethical considerations.

## Other Points and Final Remarks

Overall, the reviewers really appreciated the fact that the authors took time to discuss the comparison of their new methodology with current state-of-the-art approaches, as well as the fact that the source code for the analysis was deposited on GitHub, which will hopefully help other groups test the model on other data. The fact that the data is not publicly available, however, makes it hard to reproduce the results presented in this manuscript.

## Abbreviations

CXR: chest x-ray
